# Systematic review on the quality of randomized controlled trials from Saudi Arabia

**DOI:** 10.1016/j.conctc.2019.100441

**Published:** 2019-08-26

**Authors:** Ahmad Mamoun Rajab, Abdulmalik Hamza, Roshdi Kotaiba Aldairi, Mohamad Mahmoud Alaloush, Juliann Saquib, Nazmus Saquib

**Affiliations:** a College of Medicine, Sulaiman Al Rajhi Colleges, P.O. Box 777, Al Bukayriah, Qassim, 51941, Saudi Arabia; b College of Medicine, Qassim University, P.O. Box 6655, Buraidah, Qassim, 51452, Saudi Arabia

**Keywords:** Randomized controlled trial, CCRBT, Research quality, Saudi Arabia, ANZCTR, Australian New Zealand Clinical Trial Registry, CCRBT, Cochrane Collaboration Risk of Bias Tool, CONSORT, Consolidated Standards of Reporting Trials, ISRCTN, International Standard Randomised Controlled Trials Number, RCT, randomized controlled trial

## Abstract

**Background:**

The quality of randomized controlled trials from Saudi Arabia is unknown since most are observational studies.

**Objective:**

To determine (1) the quantity and quality of randomized controlled trials published from Saudi Arabia, and (2) whether significance of intervention effect varied by study quality.

**Methods:**

PubMed, SCOPUS, and Cochrane were searched with keywords for trials published from Saudi Arabia until February 2018. A total of 422 records were identified and screened, resulting in 61 eligible trials for analysis. Two researchers abstracted trial characteristics and assessed quality in seven domains (randomization, allocation concealment, blinding of assessors or participants, incomplete outcome data, selective reporting, and other sources of bias) using the Cochrane Collaboration Risk of Bias Tool.

**Results:**

A majority of the trials (57%) were published during 2010–2018. High risk of bias was present for blinding (outcome: 13%; participants and personnel: 28%). Biases could not be assessed due to lack of information (unclear risk) in the domains of randomization (54%), allocation concealment (44%), and blinding of outcome assessment (57%). When all seven domains were considered together (summary risk of bias), 0% of the trials had low risk, 39% had high risk, and 61% had unclear risk of biases. A greater proportion of high-risk trials had significant intervention effect than unclear-risk trials (79% vs. 67%).

**Conclusion:**

The volume and quality of trials in Saudi Arabia was low. More high-quality randomized controlled trials are warranted to address chronic diseases.

## 1 Introduction

Randomized controlled trial (RCT) is the best design available in biomedical research [1]. Randomization eliminates confounding variables and enables RCT to pinpoint the cause of a disease [2]. However, it is more frequently used to test treatments for diseases. RCT can test a new drug, a lifestyle intervention, or a procedure [3,4]. As a result, it has become the cornerstone of evidence-based medicine and is the source of most treatment guidelines [5].

RCTs are not without limitations; often, they are underpowered (inadequate sample size) [6,7], fail to implement randomization [8,9] and concealment of allocation [10] properly, and experience high loss to follow-up of participants [11]. Low-quality trials tend to produce a larger treatment effect, and the results are generally not reproducible [[12], [13], [14]].

There are three broad types of tools (i.e., item, checklist, and scale) available for the objective assessment of RCT [[15], [16], [17], [18], [19]]. The item-based tools deal with an individual component of methodological concern, for example, allocation concealment. A checklist tool consists of multiple components but without a scoring of individual items. A scale assessment tool consists of multiple components and a summary score of the items. Of these, a scale tool is more convenient to apply and facilitates inter-study comparison [16]. However, a scale tool does not address allocation concealment, assigns ‘weights’ to different items, and places more emphasis on reporting and less emphasis on actual conduct of the trial [20].

The Cochrane Collaboration Risk of Bias Tool (CCRBT) is neither a scale nor a checklist. It is a domain-based evaluation tool in which critical assessments are made separately in 7 different RCT domains (sequence generation, allocation concealment, blinding of participants and personnel, blinding of outcome assessors, incomplete outcome data, selective outcome reporting, and other sources of bias) [15]. A defect in any of these domains results in specific types of biases (selection, performance, detection, attrition, or reporting).

The volume of biomedical research in Saudi Arabia has been rapidly expanding [21]. Its research volume is second among the Arab states after Egypt [[22], [23], [24]]. However, the trend is toward publishing observational studies in local journals with a low impact factor [22]. The high prevalence of diabetes, obesity, and cardiovascular diseases among the Saudis necessitates local researchers’ engagement in extensive high-quality research (e.g., RCT) [25,26]. The overall quantity or quality of RCTs conducted in various medical fields is generally unknown. The limited data available indicates it may not be that high; for example, only 3% (n = 9) of 295 published studies on cardiovascular diseases were of experimental design [27].

Therefore, in this systematic review, we compiled all biomedical RCTs from Saudi Arabia. We described the general and specific characteristics of the RCTs, assessed their quality with CCRBT, and tested whether there was an association between trial quality and the significance of the intervention tested.

## 2 Materials and methods

The study was conducted from March to April 2018 at Sulaiman Al Rajhi Colleges in Bukairiyah, Al-Qassim province, Saudi Arabia.

*Search strategy*: We did a comprehensive search using the following databases: PubMed, SCOPUS and Cochrane Central Register of Controlled Trials (CENTRAL). Medical Subject Headings (MeSH) were used to identify RCT publications. We used the following terms: Saudi Arabia, Randomized Controlled Trial, Clinical Trial. All trials published from inception until February 1, 2018 were included.

**Fig. 1:**
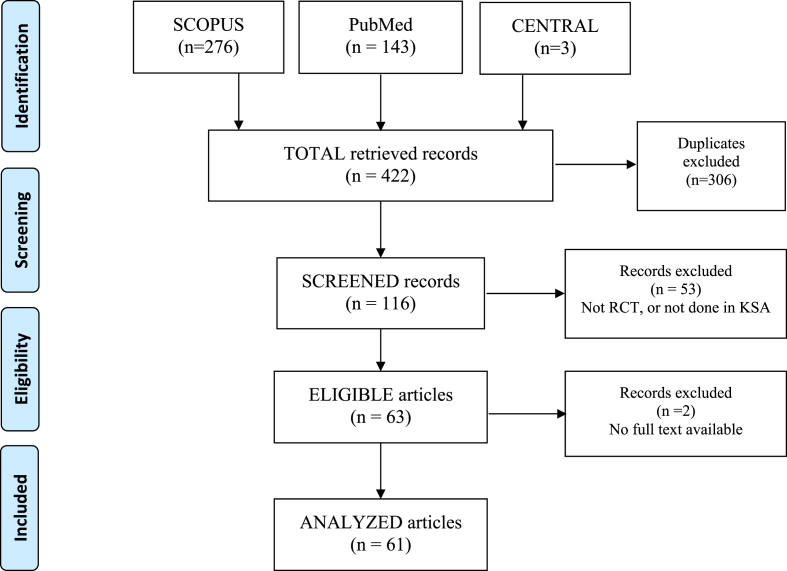
Flow chart of study eligibility among randomized controlled trials in Saudi Arabia (KSA) from 1987 to 2018 (n = 61).

*Exclusion of studies and pilot testing*: We identified a total of 422 records from SCOPUS (n = 276), PubMed (n = 143), and CENTRAL (n = 3). We removed the duplicates (n = 306) and screened the unique records (n = 116) with the following inclusion criteria: (1) an RCT design and (2) conducted, either completely in Saudi Arabia or partially (with international collaboration). We excluded 53 records (e.g., editorials, letters, and reviews), leaving 63 studies that met the eligibility criteria including 2 additional RCTs from the reference lists of the eligible studies. We could not retrieve the full text of 2 articles despite making the effort (e.g., contacting authors via email); therefore, our final analysis was limited to 61 trials (Fig. 1).

We pilot tested data extraction with 20 eligible trials. Two co-authors independently extracted the data, which we then compared to determine consistency in the coding. All researchers discussed discrepancies until we reached a consensus. We corrected the codebook and database before full data extraction.

*Data extraction*: We extracted from each trial its publication year, first author's gender, field of study, geographical location, citations, intervention type, sample size, study arms, type of participants, primary outcome, significance of primary outcome, protocol registration, funding, and international collaboration.

We also assessed each trial in light of the 7 domains of CCRBT: (1) random sequence generation, (2) allocation concealment, (3) blinding of participants and personnel, (4) blinding of outcome assessors, (5) incomplete outcome data, (6) selective outcome reporting, and (7) other sources of bias. For each domain, we categorized the trials into ‘low’, ‘high’ or ‘unclear’ risk of bias based on the criteria provided by the Cochrane Collaboration Tool. Low risk of bias meant that bias, if present, was unlikely to have altered the results. Unclear risk of bias meant that there was not enough information mentioned about key domains and that it raised some doubts about the results. High risk of bias meant that the bias might have altered the results [20].

*Statistical analysis*: We used descriptive statistics to report on frequencies, means, and standard deviations, depending on the variables concerned. We grouped the trials by publication year in 5-year blocks (≤1995, 1996–2000, 2001–2005, 2006–2010, and 2011–2018); frequency of female gender as first author, funding status, and international collaboration were calculated and plotted against the 5-year time blocks to demonstrate the change over time. We reported the risk of bias frequency for individual items of CCRBT, and calculated a summary risk of bias with the following criteria: ‘low-risk trial’ if all domains were of low risk, ‘unclear-risk trial’ if all domains were of low or unclear risk, and ‘high-risk trial’ if one or more domain was of high risk. We also calculated summary risk of bias by a second method [28]. According to this method, the definition of ‘low-risk trial’ remained the same as above, but one point was given for each domain classified as ‘unclear’ or ‘high’ risk. A trial was labeled ‘moderate-risk trial’ if the summary score was ≤2 and ‘high-risk trial’ if the score >2. We compared the likelihood of primary outcome significance across the levels of summary risk of bias. We conducted the analyses in SPSS (version 24), IBM Analytics.

## 3 Results

**Fig. 2:**
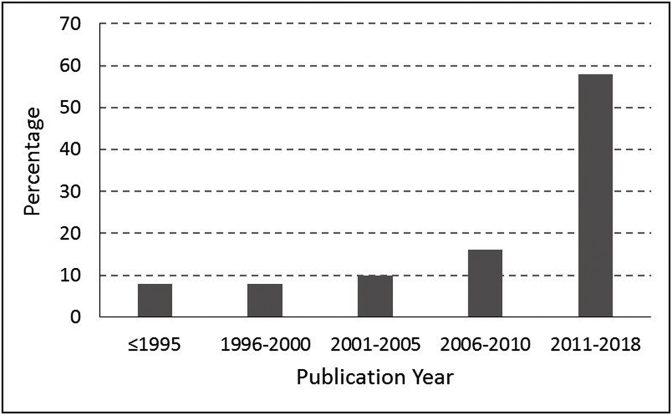
Time trend of randomized controlled trials published in Saudi Arabia from 1987 to 2018 (n = 61).

*Description of the RCTs:* The sample included 61 eligible trials (Appendix 1). The first trial was published in 1987. A total of 10 trials were published up until 2000. There were 16 trials published in 2001–2010, and 35 trials published in 2011–2018 (Fig. 2).

The predominant field of study was medicine (82%) followed by dentistry (15%); the remaining (3%) were from medical education and animal studies. The mean size of the trials was 624 (range: 15–24,226; SD: 3125). Half of the trials (51%) had a sample size under 100. The trial with the sample size of 24,226 was an international trial where Saudi Arabia was a collaborating partner [29].

Most trials were conducted in a hospital or clinic (74%). A majority of the trials had two study arms (79%) and one primary outcome (54%). The most common intervention tested was a pharmaceutical drug (44%). A majority (72%) reported a significant primary outcome. The highest number of trials came from the central region (43%), specifically from King Saud University in Riyadh, followed by the western region (36%), specifically from King Abdul-Aziz University in Jeddah (Table 1).

**Table 1:** Description of randomized controlled trials in Saudi Arabia from 1987 to 2018 (n = 61).

		COUNT	PERCENTAGE
Sample Size (mean, SD: 649, 3260) range (15–24526)			
Field of study	Medicine	50	82.0
	Dentistry	9	14.8
	Other	2	3.3
Sample source	General population	3	4.9
	Hospitals/clinics	45	73.8
	Other	13	21.3
Study arms	Two arms	48	78.7
	Three arms	11	18.0
	Four arms	2	3.3
Intervention type	Medication	27	44.3
	Behavior	10	16.4
	Surgical	7	11.5
	Other	17	27.9
Geographical location	Central region	26	42.6
	Eastern region	7	11.5
	Western region	22	36.1
	Southern region	5	8.2
	Northern region	1	1.6
Primary outcome	One outcome	33	54.1
	Two outcomes	11	18.0
	Three outcomes	12	19.7
	Four outcomes	4	6.6
	Five outcomes	1	1.6
Primary outcome significance	No	17	27.9
	Yes	44	72.1
Registered Protocol	No	51	83.6
	Yes	10	16.4

The trial numbers increased over time; the highest was observed between 2010 and 2018 (57%). Eighty-two percent of the trials (50/61) had governmental funding; the relative proportion decreased over time as private funding rose. Twenty percent of the trials (15/61) had international collaborative partners. Although the absolute number of collaborative trials increased over time, the relative proportion did not show a definitive pattern. Eleven percent of the trials (7/61) had a female as lead author; the first was in 1999 and the second in 2000, none during 2001-05 or 2006-10, and five during 2010-18.

A total of 51 trials (83.6%) did not have their protocol registered in any of the recognized registries (e.g., ANZCTR, ISRCTN, U.S. National Library of Medicine's clinicaltrials.gov, etc.), while the remaining 10 trials (16.4%) had registered protocols.

*Trial quality:* Very few trials exhibited a high risk of bias in the domain of randomization sequence generation (<2%), allocation concealment (5%), and selective reporting (<2%). A significant portion, however, had high-risk bias in blinding-related domains: outcome assessment (13%), and participants and staff (28%).

Most trials were largely free of biases (low risk) in the domain of incomplete data (87%) and selective reporting (93%). Only half were bias free in the domain of randomization sequence generation (44%), allocation concealment (51%), and blinding of participants and staff (46%). Bias could not be ascertained due to lack of information for a significant portion of the trials in the following domains: random sequence generation (54%), allocation concealment (44%), and blinding of outcome assessment (57%) (Table 2).

**Table 2:** Quality assessment using the Cochrane Collaboration Tool for assessing risk of bias among randomized controlled trials in Saudi Arabia from 1987 to 2018 (n = 61).

DOMAIN	RISK OF BIAS		
	Low Risk %	High Risk %	Unclear Risk %
Random sequence generation	44.3	1.6	54.1
Allocation concealment	50.8	4.9	44.3
Blinding of participants and personnel	45.9	27.9	26.2
Blinding of outcome assessment	29.5	13.1	57.4
Incomplete outcome data	86.9	8.2	4.9
Selective reporting	93.4	1.6	4.9
Other sources of bias	24.6	16.4	59.0
Overall risk of bias	0.0	39.3	60.7

**Fig. 3:**
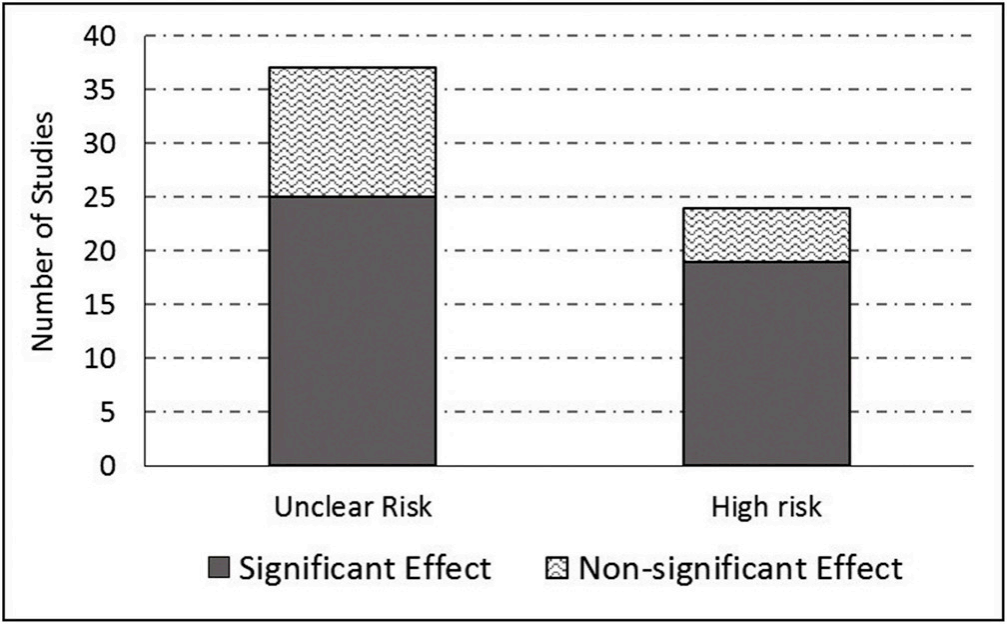
Proportion of trials with significant effects according to summary risk of bias – all trials published in Saudi Arabia from 1987 to 2018 (n = 61).

Overall, there were no ‘low-risk trials’; 60.7% were ‘unclear-risk trials’, and 39.3% were ‘high-risk trials’. High-risk trials were more likely to have found a significant effect of the tested intervention than the unclear-risk trials: 79% vs. 68% (difference was not statistically significant) (Fig. 3). Thirty-one percent (31%) were ‘moderate-risk trials’, and 69% were ‘high-risk trials’ according to the second method of summary bias calculation. High-risk trials in this method were also more likely to have found a significant effect of the tested intervention than moderate-risk trials: 79% vs. 58% (difference was not statistically significant).

## 4 Discussion

The overall number of trial publications in Saudi Arabia, to date, is small, although the number has increased over time. These trials were conducted mostly in large universities located in Riyadh and Jeddah and were funded predominantly by the government. One-fifth of the trials had an international partner, and one-tenth had a female lead author. The risk of bias was low in some domains (e.g., incomplete outcome data or selective reporting) and was high in others (e.g., blinding). Half of the trials did not provide enough information in the publication to have an assessment on risk of bias in 4 out of 7 domains of CCRBT (unclear risk).

Our findings that trial productivity increased across time and that most were conducted in large cities are supported by the literature. Between 2008 and 2012, publications from Saudi Arabia increased by an average of 14%. Of those publications, 70% originated from two universities in Riyadh and Jeddah [22]. That 11% of the publications had a female as lead author is encouraging. It reflects a substantial participation of women in academia and in the workforce [30]. Women comprise 52% of university graduates and 31% of the employees in the Ministry of Health in Saudi Arabia [31,32]. However, representation of female researchers in publication was far lower than the corresponding estimates from the developed world; as early as 2004, 37% of all publications from the UK and 29% from the USA had a female as lead author [33,34].

We found that 54% of the trial publications from Saudi Arabia did not provide details on random sequence generation to make an assessment of bias (unclear risk); this was comparable to the results of a Chinese study that reported two-thirds of its included trials failed to report details of randomization [35]. We also found details on allocation concealment missing in 44% of the trials. Similarly, 64% of the trials included in the meta-analyses had inadequate or unclear allocation concealment, and intervention effect estimates were exaggerated by 17% compared with those with adequate allocation concealment [36]. If only trials with adequate allocation concealment were included in the analyses, two-thirds of conclusions in favor of interventions were no longer supported [37].

We found that the majority of the trial publications suffered from blinding-related biases (range unclear or high risk: 50–70%), whether they were related to outcome assessments or participants/staff. In a meta-epidemiologic study, lack of or unclear double-blinding (vs. double-blinding) was associated with an average of 13% exaggeration of intervention effects [38].

We found 39% of the trial publications from Saudi Arabia had a high risk of bias, when all 7 domains of the CCRBT (summary risk of bias) were taken into account. This finding is alarming and should act as a reminder for local researchers when they design and conduct their trials. They should pay equal attention during manuscript preparation to ensure that they describe the methods clearly and in detail. This will help fellow researchers and readers to gauge the risk of bias for themselves (summary unclear risk of bias 60% in this report). We did not find any trial with a low risk of bias in all domains, which was not that unusual since other studies have reported more or less the same evaluation [28,39].

We also found that trials with a high risk of bias were more likely to produce significant results than trials with unclear (or moderate) risk of bias. The small number of trials (n = 61) may have rendered the comparison statistically insignificant. There was ample evidence that bias that came from any direction – due to small sample, inadequate random sequence generation or allocation concealment, failure to blinding – was more likely to produce significant results [36,38,40,41].

It is outside the scope of this paper to identify reasons as to why a large number of Saudi-based RCTs were of low quality. A reasonable hypothesis may be that there are not enough local researchers and fieldworkers seasoned enough to understand the complexity of designing and running a trial, especially when on the face of it, a trial seems straightforward in design and analyses. In fact, none of the Saudi universities offer a Doctor of Philosophy program in epidemiology, health behavior, or biostatistics. Another reason could be that local researchers overwhelmingly engage in explorative types of studies (e.g., cross-sectional) and therefore do not get practical training in hypothesis-testing studies [27,42].

We acknowledge several limitations of our paper. Although we searched multiple databases for eligible trials, we may have missed a few. It is, however, unlikely since trials are published in relatively higher quality journals, which are typically indexed. Our analyses took into account only published trials and not the unpublished ones. Another caveat was that risk-of-bias assessment for trials was subjective. We tried to minimize misclassification and subjectivity by having 2 co-authors abstract data independently and a third investigator adjudicating the differences.

## 5 Conclusion

The volume and quality of trials in Saudi Arabia was low. Local researchers ought to increase their effort in conducting more trials in the biomedical field, testing new therapies, finding interventions that are suitable and acceptable to people, and assessing health service procedures to make healthcare delivery in Saudi Arabia more efficient. Government funding agencies and policy makers should set higher goals for experimental studies and allocate funds accordingly. The ethical review committees in Saudi Arabia consider study methodologies in addition to ethical matters before they issue approval of the submitted proposals. The reviewers of these committees should receive trial-related methodological training so that they can help researchers develop studies with sound design and protocol. Finally, editors of local journals should ensure that researchers follow the Consolidated Standards of Reporting Trials (CONSORT) when they submit trials for publication.

## Declaration of conflicting interest

The authors declare that they have no conflicts of interest related to this work.

## Funding

This research did not receive any specific grant from funding agencies in the public, commercial, or not-for-profit sectors.
