# Supplementary material for: Systematic review on the quality of randomized controlled trials from Saudi Arabia
Source: Contemp Clin Trials Commun. 2019 Aug 26;16:100441. doi: 10.1016/j.conctc.2019.100441 (PMC6737301; doi:10.1016/j.conctc.2019.100441)
Supplement: Multimedia component 1 [file mmc1.docx]

**Appendix A - List of randomized controlled trials included (n=61):**

(1-61)

[1] Nichol A, French C, Little L, Haddad S, Presneill J, Arabi Y, et al. Erythropoietin in traumatic brain injury (EPO-TBI): a double-blind randomised controlled trial. ***Lancet*** 2015;386(10012):2499-506.

[2] Aboalshamat K, Hou XY, Strodl E. The impact of a self-development coaching programme on medical and dental students' psychological health and academic performance: a randomised controlled trial. ***BMC Med Educ*** 2015;15:134.

[3] AlBedah A, Khalil M, Elolemy A, Hussein AA, AlQaed M, Al Mudaiheem A, et al. The use of wet cupping for persistent nonspecific low back pain: randomized controlled clinical trial. ***J Altern Complement Med*** 2015;21(8):504-8.

[4] Alsohaibani F, Al Ashgar H, Al Kahtani K, Kagevi I, Peedikayil M, Alfadda A, et al. Prospective trial in Saudi Arabia comparing the 14-day standard triple therapy with the 10-day sequential therapy for treatment of Helicobacter pylori infection. ***Saudi J Gastroenterol*** 2015;21(4):220-5.

[5] Khan MU, Aqil M, Hussain A, Zahrani TA, Hillis M. Comparison of the effect of pre-operative single oral dose of tramadol and famotidine on gastric secretions pH and volume in patients scheduled for laparoscopic cholecystectomy. ***J Coll Physicians Surg Pak*** 2015;25(5):320-3.

[6] Khalil M, Al-Mazrou Y, Findlow H, Chadha H, Bosch Castells V, Oster P, et al. Meningococcal serogroup C serum and salivary antibody responses to meningococcal quadrivalent conjugate vaccine in Saudi Arabian adolescents previously vaccinated with bivalent and quadrivalent meningococcal polysaccharide vaccine. ***Vaccine*** 2014;32(43):5715-21.

[7] Youssef A. Use of short message service reminders to improve attendance at an internal medicine outpatient clinic in Saudi Arabia: a randomized controlled trial. ***East Mediterr Health J*** 2014;20(5):317-23.

[8] Othman I, Hady HA. Hernia sac of indirect inguinal hernia: invagination, excision, or ligation? ***Hernia*** 2014;18(2):199-204.

[9] Muhammad MI. Thoracoscopic repair of pectus excavatum using different bar stabilizers versus open repair. ***Asian Cardiovasc Thorac Ann*** 2014;22(2):187-92.

[10] Rouzi AA, Alsibiani S, Mansouri N, Alsinani N, Darhouse K. Randomized clinical trial between hourly titrated oral misoprostol and vaginal dinoprostone for induction of labor. ***Am J Obstet Gynecol*** 2014;210(1):56.e1-6.

[11] Mohanlall R, Adam J, Nemlander A. Venoarterial modified ultrafiltration versus conventional arteriovenous modified ultrafiltration during cardiopulmonary bypass surgery. ***Ann Saudi Med*** 2014;34(1):18-30.

[12] Aldaqal SM, Kensarah AA, Alhabboubi M, Ashy AA. A new technique in management of pilonidal sinus, a university teaching hospital experience. ***Int Surg*** 2013;98(4):304-6.

[13] Al-Baghli NA. Evidence based medicine workshop. Randomized controlled trial of the efficacy on physician's knowledge and skills. ***Saudi Med J*** 2013;34(10):1055-61.

[14] Abd El-Kader MS, Al-Jiffri O, Ashmawy EM. Impact of weight loss on markers of systemic inflammation in obese Saudi children with asthma. ***Afr Health Sci*** 2013;13(3):682-8.

[15] Borja-Tabora C, Montalban C, Memish ZA, Van der Wielen M, Bianco V, Boutriau D, et al. Immune response, antibody persistence, and safety of a single dose of the quadrivalent meningococcal serogroups A, C, W-135, and Y tetanus toxoid conjugate vaccine in adolescents and adults: results of an open, randomised, controlled study. ***BMC Infect Dis*** 2013;13:116.

[16] Hussain MI, Alam MK, Al-Qahatani HH, Al-Akeely MH. Role of postoperative antibiotics after appendectomy in non-perforated appendicitis. ***J Coll Physicians Surg Pak*** 2012;22(12):756-9.

[17] Shohayeb A, El-Khayat W. Does a single endometrial biopsy regimen (S-EBR) improve ICSI outcome in patients with repeated implantation failure? A randomised controlled trial. ***Eur J Obstet Gynecol Reprod Biol*** 2012;164(2):176-9.

[18] Al-Mazrou Y, Khalil M, Findlow H, Chadha H, Bosch Castells V, Johnson DR, et al. Immunogenicity and safety of a meningococcal quadrivalent conjugate vaccine in Saudi Arabian adolescents previously vaccinated with one dose of bivalent and quadrivalent meningococcal polysaccharide vaccines: a phase III, controlled, randomized, and modified blind-observer study. ***Clin Vaccine Immunol*** 2012;19(7):999-1004.

[19] Almarakbi WA, Alhashemi JA, Kaki AM. Adding a conduit to GlideScope blade facilitates tracheal intubation: prospective randomized study. ***Saudi Med J*** 2012;33(6):617-21.

[20] Al Moamary MS, Al-Kordi AG, Al Ghobain MO, Tamim HM. Utilization and responsiveness of the asthma control test (ACT) at the initiation of therapy for patients with asthma: a randomized controlled trial. ***BMC Pulm Med*** 2012;12:14.

[21] Mohamed WA, Ismail M. A randomized, double-blind, prospective study of caspofungin vs. amphotericin B for the treatment of invasive candidiasis in newborn infants. ***J Trop Pediatr*** 2012;58(1):25-30.

[22] Mutwalli HA, Fallows SJ, Arnous AA, Zamzami MS. Randomized controlled evaluation shows the effectiveness of a home-based cardiac rehabilitation program. ***Saudi Med J*** 2012;33(2):152-9.

[23] Alamoudi NM, Hanno AG, Sabbagh HJ, Masoud MI, Almushayt AS, El Derwi DA. Impact of maternal xylitol consumption on mutans streptococci, plaque and caries levels in children. ***J Clin Pediatr Dent*** 2012;37(2):163-6.

[24] Khan MN, Telmesani A, Alkhotani A, Elzouki A, Edrees B, Alsulimani MH. Comparison of jeopardy game format versus traditional lecture format as a teaching methodology in medical education. ***Saudi Med J*** 2011;32(11):1172-6.

[25] Sofrata A, Brito F, Al-Otaibi M, Gustafsson A. Short term clinical effect of active and inactive Salvadora persica miswak on dental plaque and gingivitis. ***J Ethnopharmacol*** 2011;137(3):1130-4.

[26] Baslaim G, Jamjoom A, Al-Githmi I, Al-Malki F. Nitroglycerin cardioplegia effect on coronary artery targets in bypass grafting. ***Thorac Cardiovasc Surg*** 2011;59(7):411-5.

[27] Baeshen HA, Lingström P, Birkhed D. Effect of fluoridated chewing sticks (Miswaks) on white spot lesions in postorthodontic patients. ***Am J Orthod Dentofacial Orthop*** 2011;140(3):291-7.

[28] El-Tahan MR, El Ghoneimy YF, Regal MA, El Emam H. Comparative study of the non-dependent continuous positive pressure ventilation and high-frequency positive-pressure ventilation during one-lung ventilation for video-assisted thoracoscopic surgery. ***Interact Cardiovasc Thorac Surg*** 2011;12(6):899-902.

[29] Abdel Aal M, ElNahal N, Bakir BM, Fouda M. Mini-cardiopulmonary bypass impact on blood conservation strategy in coronary artery bypass grafting. ***Interact Cardiovasc Thorac Surg*** 2011;12(4):600-4.

[30] Memish ZA, Dbaibo G, Montellano M, Verghese VP, Jain H, Dubey AP, et al. Immunogenicity of a single dose of tetravalent meningococcal serogroups A, C, W-135, and Y conjugate vaccine administered to 2- to 10-year-olds is noninferior to a licensed-ACWY polysaccharide vaccine with an acceptable safety profile. ***Pediatr Infect Dis J*** 2011;30(4):e56-62.

[31] Arabi YM, Dabbagh OC, Tamim HM, Al-Shimemeri AA, Memish ZA, Haddad SH, et al. Intensive versus conventional insulin therapy: a randomized controlled trial in medical and surgical critically ill patients. ***Crit Care Med*** 2008;36(12):3190-7.

[32] Menzies D, Long R, Trajman A, Dion MJ, Yang J, Al Jahdali H, et al. Adverse events with 4 months of rifampin therapy or 9 months of isoniazid therapy for latent tuberculosis infection: a randomized trial. ***Ann Intern Med*** 2008;149(10):689-97.

[33] Arabi YM, Aljumah A, Dabbagh O, Tamim HM, Rishu AH, Al-Abdulkareem A, et al. Low-dose hydrocortisone in patients with cirrhosis and septic shock: a randomized controlled trial. ***CMAJ*** 2010;182(18):1971-7.

[34] Shokry M, Manaa EM, Shoukry RA, Shokeir MH, Elsedfy GO, Abd El-Aziz Al-S. Effects of intrapartum epidural analgesia at high altitudes: maternal, fetal, and neonatal outcomes. A randomized controlled trial of two formulations of analgesics. ***Acta Obstet Gynecol Scand*** 2010;89(7):909-15.

[35] Al Faleh HF, Thalib L, AlHabib KF, Ullah A, AlNemer K, AlSaif SM, et al. Are acute coronary syndrome patients admitted during off-duty hours treated differently? An analysis of the Saudi Project for Assessment of Acute Coronary Syndrome (SPACE) study. ***Ann Saudi Med*** 2012;32(4):366-71.

[36] Sonbul H, Birkhed D. The preventive effect of a modified fluoride toothpaste technique on approximal caries in adults with high caries prevalence. A 2-year clinical trial. ***Swed Dent J*** 2010;34(1):9-16.

[37] Makhdoom NK, Farid MF. Prophylactic antiemetic effects of midazolam, dexamethasone, and its combination after middle ear surgery. ***Saudi Med J*** 2009;30(4):504-8.

[38] Eskandar M, Abou-Setta AM, Almushait MA, El-Amin M, Mohmad SE. Ultrasound guidance during embryo transfer: a prospective, single-operator, randomized, controlled trial. ***Fertil Steril*** 2008;90(4):1187-90.

[39] Al-Mulhim AS, Ali AM, Al-Masuod N, Alwahidi A. Post hemorrhoidectomy pain. A randomized controlled trial. ***Saudi Med J*** 2006;27(10):1538-41.

[40] Alfaleh FZ, Hadad Q, Khuroo MS, Aljumah A, Algamedi A, Alashgar H, et al. Peginterferon alpha-2b plus ribavirin compared with interferon alpha-2b plus ribavirin for initial treatment of chronic hepatitis C in Saudi patients commonly infected with genotype 4. ***Liver Int*** 2004;24(6):568-74.

[41] Al-Traif I, Handoo FA, Al-Jumah A, Al-Nasser M. Chronic hepatitis C. Genotypes and response to anti-viral therapy among Saudi patients. ***Saudi Med J*** 2004;25(12):1935-8.

[42] Samarkandi AH, Shaikh MA, Ahmad RA, Alammar AY. Use of dexamethasone to reduce postoperative vomiting and pain after pediatric tonsillectomy procedures. ***Saudi Med J*** 2004;25(11):1636-9.

[43] Al-Otaibi M, Al-Harthy M, Söder B, Gustafsson A, Angmar-Månsson B. Comparative effect of chewing sticks and toothbrushing on plaque removal and gingival health. ***Oral Health Prev Dent*** 2003;1(4):301-7.

[44] Alrajhi AA, Ibrahim EA, De Vol EB, Khairat M, Faris RM, Maguire JH. Fluconazole for the treatment of cutaneous leishmaniasis caused by Leishmania major. ***N Engl J Med*** 2002;346(12):891-5.

[45] Villar J, Ba'aqeel H, Piaggio G, Lumbiganon P, Miguel Belizán J, Farnot U, et al. WHO antenatal care randomised trial for the evaluation of a new model of routine antenatal care. ***Lancet*** 2001;357(9268):1551-64.

[46] Khalil MK, al-Mazrou YY, al-Ghamdi YS. Vaccines: World Health Organization versus Federal Drug Administration recommended formula. ***East Mediterr Health J*** 2000;6(4):644-51.

[47] Bahijri SM. Effect of chromium supplementation on glucose tolerance and lipid profile. ***Saudi Med J*** 2000;21(1):45-50.

[48] Farsi NM. The effect of education upon dentists' knowledge and attitude toward fissure sealants. ***Odontostomatol Trop*** 1999;22(86):27-32.

[49] Al-Faris E, Al-Subaie A, Khoja T, Al-Ansary L, Abdul-Raheem F, Al-Hamdan N, et al. Training primary health care physicians in Saudi Arabia to recognize psychiatric illness. ***Acta Psychiatr Scand*** 1997;96(6):439-44.

[50] Tabbara KF, Abu-el-Asrar A, al-Omar O, Choudhury AH, al-Faisal Z. Single-dose azithromycin in the treatment of trachoma. A randomized, controlled study. ***Ophthalmology*** 1996;103(5):842-6.

[51] Abolfotouh MA. The impact of a lecture on AIDS on knowledge, attitudes and beliefs of male school-age adolescents in the Asir Region of southwestern Saudi Arabia. ***J Community Health*** 1995;20(3):271-81.

[52] al-Quorain A, Larbi EB, al-Shedoki F. A double-blind, randomized, placebo-controlled trial of cisapride in Saudi Arabs with functional dyspepsia. ***Scand J Gastroenterol*** 1995;30(6):531-4.

[53] Johansen K, Woodhouse NJ, Odugbesan O. Comparison of 1073 MBq and 3700 MBq iodine-131 in postoperative ablation of residual thyroid tissue in patients with differentiated thyroid cancer. ***J Nucl Med*** 1991;32(2):252-4.

[54] Schwartz B, Al-Tobaiqi A, Al-Ruwais A, Fontaine RE, A'ashi J, Hightower AW, et al. Comparative efficacy of ceftriaxone and rifampicin in eradicating pharyngeal carriage of group A Neisseria meningitidis. ***Lancet*** 1988;1(8597):1239-42.

[55] Karrar ZA, Abdulla MA, Moody JB, Macfarlane SB, Al Bwardy M, Hendrickse RG. Loperamide in acute diarrhoea in childhood: results of a double blind, placebo controlled clinical trial. ***Ann Trop Paediatr*** 1987;7(2):122-7.

[56] S GN, Kamal W, George J, Manssor E. Radiological and biochemical effects (CTX-II, MMP-3, 8, and 13) of low-level laser therapy (LLLT) in chronic osteoarthritis in Al-Kharj, Saudi Arabia. ***Lasers Med Sci*** 2017;32(2):297-303.

[57] Javed F, Abduljabbar T, Carranza G, Gholamiazizi E, Mazgaj DK, Kellesarian SV, et al. Efficacy of periimplant mechanical debridement with and without adjunct antimicrobial photodynamic therapy in the treatment of periimplant diseases among cigarette smokers and non-smokers. ***Photodiagnosis Photodyn Ther*** 2016;16:85-9.

[58] Babiker EE, Al Juhaimi F, Ghafoor K, Mohamed HE, Abdoun KA. Effect of partial replacement of alfalfa hay with Moringa species leaves on milk yield and composition of Najdi ewes. ***Trop Anim Health Prod*** 2016;48(7):1427-33.

[59] Mohammed M, Eggers SM, Alotaiby FF, de Vries N, de Vries H. Effects of a randomized controlled trial to assess the six-months effects of a school based smoking prevention program in Saudi Arabia. ***Prev Med*** 2016;90:100-6.

[60] Sonbul H, Merdad K, Birkhed D. The effect of a modified fluoride toothpaste technique on buccal enamel caries in adults with high caries prevalence: a 2-year clinical trial. ***Community Dent Health*** 2011;28(4):292-6.

[61] Al-Hwiesh AK. A modified peritoneal dialysis catheter with a new technique: Farewell to catheter migration. ***Saudi J Kidney Dis Transpl*** 2016;27(2):281-9.
